# Novel aspects of age-protection by spermidine supplementation are associated with preserved telomere length

**DOI:** 10.1007/s11357-020-00310-0

**Published:** 2021-01-31

**Authors:** Alexander Wirth, Bettina Wolf, Cheng-Kai Huang, Silke Glage, Sebastian J. Hofer, Marion Bankstahl, Christian Bär, Thomas Thum, Kai G. Kahl, Stephan J. Sigrist, Frank Madeo, Jens P. Bankstahl, Evgeni Ponimaskin

**Affiliations:** 1 Cellular Neurophysiology, Hanover Medical School, Carl-Neuberg-Straße 1, 30625 Hannover, Germany; 2 Preclinical Molecular Imaging, Department of Nuclear Medicine, Hanover Medical School, Carl-Neuberg-Straße 1, 30625 Hannover, Germany; 3 Institute of Molecular and Translational Therapeutic Strategies, Hanover Medical School, Carl-Neuberg-Straße 1, 30625 Hannover, Germany; 4 Institute for Laboratory Animal Science, Hanover Medical School, Carl-Neuberg-Straße 1, 30625 Hannover, Germany; 5 Institute of Molecular Biosciences, Karl-Franzens-Universität Graz, Humboldtstraße 50/EG, 8010 Graz, Austria; 6 REBIRTH Center for Translational Regenerative Medicine, Hanover Medical School, Carl-Neuberg-Straße 1, 30625 Hannover, Germany; 7 Fraunhofer Institute for Toxicology and Experimental Medicine (ITEM), Nikolai-Fuchs-Straße 1, 30625 Hannover, Germany; 8 Dept. of Psychiatry; Social Psychiatry and Psychotherapy, Hanover Medical School, Carl-Neuberg-Straße 1, 30625 Hannover, Germany; 9 Freie University Berlin, Institute of Biology, Takusstraße 6, 14195 Berlin, Germany; 10 Institute of Neuroscience, Lobachevsky State University of Nizhny Novgorod, Gagarin ave. 23, Nizhny Novgorod, Russian Federation 603950

**Keywords:** Spermidine, Ageing, Hair growth, Glucose metabolism, PET, Cardiac telomeres

## Abstract

Ageing provokes a plethora of molecular, cellular and physiological deteriorations, including heart failure, neurodegeneration, metabolic maladaptation, telomere attrition and hair loss. Interestingly, on the molecular level, the capacity to induce autophagy, a cellular recycling and cleaning process, declines with age across a large spectrum of model organisms and is thought to be responsible for a subset of age-induced changes. Here, we show that a 6-month administration of the natural autophagy inducer spermidine in the drinking water to aged mice is sufficient to significantly attenuate distinct age-associated phenotypes. These include modulation of brain glucose metabolism, suppression of distinct cardiac inflammation parameters, decreased number of pathological sights in kidney and liver and decrease of age-induced hair loss. Interestingly, spermidine-mediated age protection was associated with decreased telomere attrition, arguing in favour of a novel cellular mechanism behind the anti-ageing effects of spermidine administration.

**Supplementary Information:**

The online version contains supplementary material available at 10.1007/s11357-020-00310-0.

## Introduction

As average human life expectancy increases constantly, understanding the mechanisms underlying physiological ageing has gained a lot of scientific interest [20]. Ageing is a natural process characterised by a progressive impairment of cellular functions leading to a time-dependent functional decline in tissue and organ homeostasis. Pathological changes in numerous cellular processes, including mitochondrial dysfunction, telomere shortening, dysregulation of nutrient-sensing and affected intracellular signalling pathways, are characteristic features of ageing in most organisms [31]. Among the many deteriorating cellular processes in ageing, autophagy plays a major role, as its restoration has been shown to counteract manifold ageing phenotypes and is capable of prolonging health and lifespan in various model organisms. Thus, autophagy-inducing interventions have become a major field of gerontology research. Overall, ageing-related deteriorations at the cellular level arguably represent the major risk factor for most non-communicable diseases [23].

Ageing however is a multifaceted process, taking place at multiple molecular, cellular and organismal levels, with organs and specific tissues being affected to a different degree. In this regard, it is becoming increasingly apparent that the brain is particularly vulnerable and highly susceptible to pathological age-related changes, making advanced age the major risk factor for developing neurodegenerative diseases. Accordingly, the number of the individuals affected by ageing-associated neurodegenerative brain disorders, importantly Alzheimer’s disease, is constantly increasing during the last decades [2], with dementia-like diseases meanwhile ranking among the top five causes of death [66].

In addition to neurodegenerative disorders, ageing represents the dominant risk factor for the development of cardiovascular diseases as well. Ageing produces numerous changes in the human heart at the molecular, structural and functional level [12]. The most common age-related alterations in the heart are cardiac hypertrophy (in particular affecting left ventricle), fibrosis and maladaptive remodelling leading to diastolic dysfunction and heart failure [54].

To date, the most robust intervention towards an increase in the healthy lifespan is caloric restriction without malnutrition [27]. However, the compliance of caloric restriction in human is low as it is rarely compatible with most people’s daily life. Thus, food supplements acting as caloric restriction mimetics (CRM) might provide attractive alternatives [32, 35, 40]. Particularly, the naturally occurring polyamine spermidine has been shown to extend life- and health span in worms, flies and mice [30, 56]. Levels of endogenous spermidine decline with age in model organisms and in humans (for review, see Madeo et al. [34]). Spermidine action has been linked to the modulation of reactive oxygen species, DNA replication, transcription and translation, anti-inflammatory properties, altered mitochondrial function, improved proteostasis, increased hypusination and the induction of autophagy [13, 37, 46, 69]. Furthermore, analysis in ageing mice has shown that spermidine feeding not only prolongs the lifespan but also exerts cardio-protective effects as well as protective measures on the synaptic and mitochondrial status of ageing mice [12, 34, 36].

In this study, we aimed to analyse potentially beneficial impact of prolonged spermidine administration on commonly age-affected organ systems, including the heart, the liver, the kidney and the brain. Therefore, we analysed the putative effects of spermidine supplementation in two cohorts of aged mice comparing animals after 6 months of supplementation, starting at an advanced age of 17/18 months, with age-matched non-treated mice. We also analysed an additional cohort of 6-month-old non-treated mice as ‘young controls’. Our results demonstrate a protective role of spermidine consumption against age-related hair loss. In addition, analysis of the metabolic changes in the brain using [^18^F]fluoro-2-deoxy-d-glucose positron emission tomography ([^18^F]FDG-PET) revealed that spermidine-treatment affected glucose uptake in the brain of aged mice towards the level observed in young animals. Furthermore, we observed cardio-protective effects of spermidine-treatment at histological levels, which were accompanied by a decrease in telomere attritionin in comparison to untreated aged animals. These results further underline the potential of spermidine supplementation to impact age-induced deteriorations of the brain, the liver, the kidney and the heart.

## Material and methods

### Animals

Male C57Bl/6JRj mice were purchased from Janvier labs at an age of either 6 months or 17 months. The 6-month-old group served as young control cohort and received no spermidine supplementation. The 17-month-old mice were randomised into two groups: one group was given drinking water supplemented with 3 mM spermidine (spd^+^) (Sigma-Aldrich, aqueous stock solution, pH 7.2) ad libitum over the whole test period. The other group received normal tap water. The initial number of animals each group was 11 according to power analyses [15] (G*power; *n* = 11, effect size = 1.5, *α* = 0.05, 1-β = 0.95). All groups were kept at a 14/10 light-dark-cycle and standard chow containing 22.5% protein, 5.1% fat, 4.5% fibre and 6.1% ash (Altromin #1314). In general, four mice were housed per cage. Of note, mice were separated into single cages at least 3 weeks prior to the final experiments to avoid barbering. PET experiments were conducted at an age of 6 or 23 months, whereas the histology sampling took place at an age of 24/25 months. Husbandry and procedures involving animals were carried out according to the German Animal Welfare Legislation as set forth by the European Convention for the Protection of Vertebrate Animals used for Experimental and Other Scientific Purposes, Council of Europe, ETS no.123, appendix VIII and according to national regulations and standards. All experiments were approved by the local Institutional Animal Care and Research Advisory Committee and by the local government, namely the Lower Saxony State Ministry of Food, Agriculture and Consumer Protection in consultation with the Animal Protection Committee with the approval ID: 33.12-42502-04-16/2206.

### Cohort parameters

Each mouse was weighed once at the age of 17 month and at the age of 23 month using a table balance (CM 320-1N, Kern). The amount of consumed water was measured within a 2-week long-lasting time period at the end of the experimental phase. The amount of water was measured twice a week for each mouse. To quantify the fur coverage, images of the mice (top view) were taken during the final experiment. Those images were converted into 8-bit files and analysed using FIJI. First, the area of the visible body (top view; excluding ears, claws, tail) was quantified. Second, the fur-uncovered area was quantified by drawing a ROI manually around the less hairy part of the body. Total area was set to 100% and fur-uncovered area was quantified in relation to the whole body.

### PET imaging

PET images were acquired using a small animal PET/computer tomography (CT) camera (Inveon, Siemens). Mice were anesthetised with 1.5–2.5% isoflurane in humidified oxygen. Monitoring of respiration (BioVet, m2m imaging) was used to adjust anaesthesia levels to maintain a stable respiration rate. Mice were positioned prone in a continuously warmed double mouse chamber (Minerve) with the brain in the centre field of view. [^18^F]FDG was injected via a custom-made catheter inserted into a lateral tail vein (12.82 ± 0.71 MBq). The scan was started with the beginning of the radio-tracer injection and dynamic data were acquired for 60 min in 32 frames (5 × 2 s, 4 × 5 s, 3 × 10 s, 8 × 30 s, 5 × 60 s, 4 × 300 s and 3 × 600 s). For image reconstruction, iterative ordered subset expectation maximization algorithm followed by 18 iterations of maximum a posteriori (OSEM3D/fastMAP) applying standard corrections for decay, random and scatter was used. For attenuation correction we referred to a 20 min ^57^Co transmission scan. After PET, a low-dose CT scan was performed to provide anatomical information for image analysis. Directly after induction of anaesthesia and at the end of the imaging session, blood glucose levels were measured by a micro puncture of the saphenous vein (Conrour XT®, Bayer Consumer Care) and averaged for image analysis. In total, 22 mice were subjected to [^18^F]FDG PET (young: *n* = 9; aged: *n* = 6; aged + spd^+^: *n* = 6). One mouse of the aged group died during the scan, and due to movement artefacts at the beginning of the scan in one mouse of the aged + spd^+^ group, only uptake analysis was possible. The whole experiment including analyses was performed in a blinded fashion.

### PET image analysis

Imaging data were analysed using PMOD software (PMOD 3.703). A region of interest was defined for the left ventricular myocardium, and for brain analysis, images were co-registered to an MRI-based volumes of interest (VOI) –atlas [38]. Uptake was analysed in minutes 50–60 after tracer injection as percent injected dose per cubic centimetre of tissue [%ID/cc]. In addition, kinetic modelling was performed. Image input function of the blood was derived from a 18 mm^3^ cylindrical VOI placed on the inferior vena cava superior to the renal branches, excluding the last three time frames. For regional brain analysis, blood time activity curves (TAC) were fitted to a two-tissue compartment model setting t4 to 0. Additionally, curves were fitted to the Patlak kinetic model. Brain influx rate constant *K*_i_ [ml/g/min] was the graphically defined Patlak slope, and the metabolic rate of glucose uptake MR_Glu_ [μmol/min/100 g] was calculated as *K*_i_ × blood glucose / LC, whereas LC is the lumped constant equally 0.67 as estimated for rodents [59]. Both models revealed highly similar results (data not shown). Additional parametric maps using Patlak graphical analysis on a voxel level were calculated with PMOD.

Average images and statistical parametric mapping (SPM) were calculated using the MATLAB software (The MathWorks) and SPM12 (University College London). For SPM, differences between groups were calculated by unpaired 2-sample *t* tests with a significance level threshold of 0.05 (uncorrected for multiple comparisons). Minimum voxel cluster size was set to 50 and threshold of *t* maps was set according to the degrees of freedom of the comparison.

### Preparations of tissue paraffin sections

At the dedicated time points, the animals were sacrificed. Tissues were fixed in neutral buffered 4% paraformaldehyde for at least 24 h. After trimming according to the Registry of Industrial Toxicology Animal-Data recommendation and dehydration (Shandon Hypercenter, XP), the samples were embedded in paraffin (TES, Medite). Sections (2–3 μm thick, microtome Reichert-Jung 2030) were deparaffinised in xylene and haematoxylin and eosin (H&E) stained or periodic acid Schiff stained according to standard protocols. Blinded evaluation of 24 mice (young: *n* = 7; aged: *n* = 9; aged spd^+^: *n* = 8) by light microscopy (Axioskop 40, Zeiss) was performed by a trained pathologist and representative microphotographs were taken (AxioCam MRc, Zeiss).

### Histology of tissue sections

The hearts were screened histologically for age-related changes. A longitudinal section through both ventricles was made from the heart base to the apex, presenting the auricles, main and surrounding vessels, aortic valve, mitral valve and in some animals as well the tricuspid valve. Samples were reviewed for signs of cardiomyopathy and endocarditis including arteritis of aorta and surrounding vessels, heart valve inflammation and atrial thrombosis. Gross organ systems were screened for neoplastic changes. For scoring, a semi-quantitative system was used. Specifically, three parameters were scored for alterations of the heart valves and of the aorta: 1, mild inflammatory infiltration; 2, marked inflammatory infiltration; 3, severe inflammatory infiltration with thrombus formation. Scores for heart valves and aorta were summarised. Neoplastic changes were reported as yes/no. The presence of a neoplastic change was scored with 1 and quantified separately.

The kidneys were analysed in respect to chronic progressive nephropathy, glomerulonephritis and amyloidosis and each part was scored according to severity [11, 43, 68]. The liver score was based on the frequency of the appearance of (a) lymphatic infiltration of the intrahepatic bile ducts, (b) parenchymla granuloma and (c) necrotising hepatitis [16, 39].

### Quantitative fluorescence in situ hybridization

Mouse cardiac tissue samples were also used for telomere analysis. Five-micrometre thick sections of paraffin-embedded tissue were cut and deparaffinised in xylene and then rehydrated in serial ethanol concentrations. The slides were washed in PBS for 5 min and incubated at 37 °C for 10 min in acidified pepsin solution (1 mg/ml pepsin, Roth; 0.01 M HCl, Roth). Slides were washed with PBS and fixed with 4% paraformaldehyde again, and then dehydrated in a 70–100% ethanol series (5 min each). After 10 min of air drying, 30 μl of telomere probe mix (10 mM Tris pH 7.4, 25 mM MgCl_2_, 9 mM citric acid, 82 mM Na_2_HPO_4_, 70% deionised formamide (Ambion), 0.25% blocking reagent (Roche) and 0.5 μg/ml Telomeric Cy3 PNA probe (Panagene)) were added to each slide. A coverslip was added and then slides were incubated at 85 °C for 3 min, and further incubated at room temperature for 2 h in a wet chamber in the dark. After probe incubation, slides were washed 2 × 15 min in wash buffer (10 mM Tris pH 7.2, 0.1% BSA and 70% formamide (Roth)) under vigorous shaking (550 rpm), then 3 × 5 min in TBS-0.08% Tween20, and then incubated with 4′,6-diamidino-2-phenylindole (DAPI) (Sigma) before mounting samples in Vectashield (Vector™). Confocal images were acquired as z-stacks every 1 μm for a total of 5 μm using a Zeiss LSM 780 confocal microscope equipped with a × 40 objective. Maximum projections were created using the ZEN software. Telomere signal intensity was quantified using the ImageJ plugin Telometer. Three sections per heart were imaged in a blinded fashion and 40 nuclei were counted for each section resulting in 120 nuclei per animal (young: *n* = 5; aged: *n* = 4; aged spd^+^: *n* = 4). To digitally reduce noise signals from non-specific qFISH probes, intensity value threshold was set to 25.000 and afterwards data were analysed for outliers using ROUT (*Q* = 1%).

### Statistical analysis

Statistical analysis was performed using the Prism7 (GraphPad Software) software. Group data were either compared by one-way analysis of variance (ANOVA) followed by Tukey’s post hoc test or Kruskal-Wallis with Dunn’s comparison for multiple comparisons. Two groups were compared using Mann Whitney test or unpaired 2-sample *t* test. Values of *p* < 0.05 were considered to be significant. If not stated otherwise, data are given as mean ± standard deviation (SD).

## Results

### Spermidine treatment protects against ageing-induced hair loss

In the present study, we investigated the role of spermidine supplementation on different aspects of the ageing process using mice as a model. To this end, we compared three independent cohorts of mice: a non-treated young control group (6 months), non-treated aged (25 months) mice and spermidine-treated aged (25 months) mice. The latter had received a 6-month long spermidine supplementation in the drinking water (3 mM, ad libitum) prior to the first time point of the analysis, starting the supplementation at 18 months of age.

**Fig. 1:**
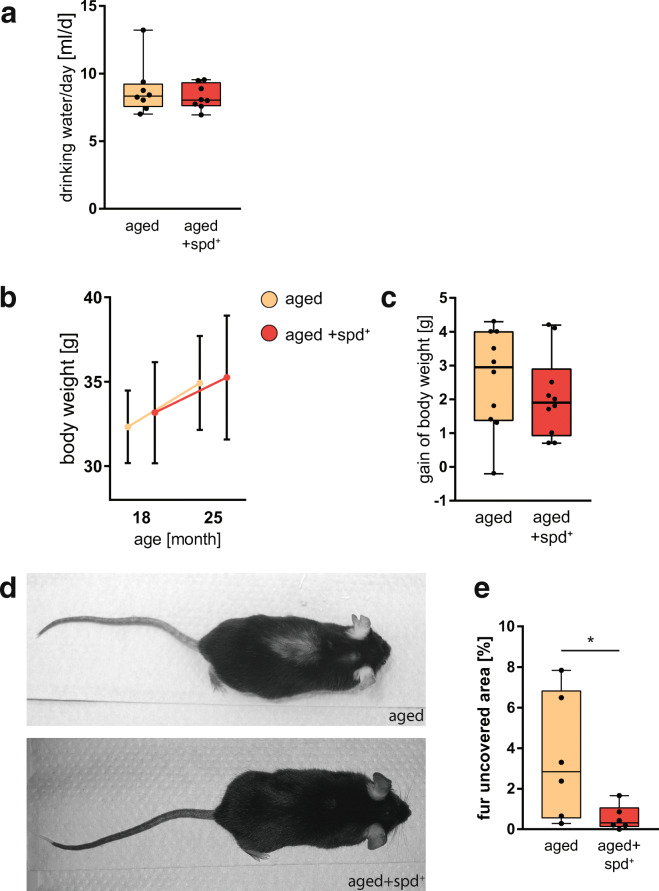
**a** Consumption of drinking water in two independent cohorts of mice including non-treated aged (25 months) and spermidine-treated (spd^+^) aged (25 months) mice. The latter was undergoing a 6-month long spermidine treatment (3 mM) in the drinking water ad libitum, starting at 18 months of age. Min-to-max box whisker of the mean water intake per day for a single mouse in both groups is shown. **b** Changes in the absolute body weight (mean + SD) in mice during ageing and treatment. **c** Difference in gain of body weight in both aged groups of mice as min-to-max box whisker plot. *N* = 10 mice per group. **d** Representative images of aged and aged+spd^+^ mice at an age of 25 month highlighting the differences in top-side fur coverage in aged animals. **e** Quantification of the hairless area in percent, depicted as min-to-max box whisker plot. *N* = 6 mice, Mann Whitney test, *p* = 0.041

First, comparing the two groups of aged animals revealed no difference in drinking behaviour, suggesting no apparent adverse effects of the administration of spermidine (spd^+^) (Fig. 1a). Moreover, both aged groups showed a similar body weight increase relative to starting point of the analysis at 18 months (Fig. 1b), with no significant differences in body weight gained (Fig. 1c). Despite these similarities, we found that aged spd^+^ animals obviously exhibited less hair loss. As shown in Fig. 1d, the spermidine-treated mice appeared to still bear fully covered bodies. In contrast, the control animals showed areas on their back with significantly sparse fur coverage (Fig. 1d). As hair loss is a characteristic feature of advanced ageing in mice [21], we quantified the effects of spermidine treatment for this phenotype. Indeed, quantitative analysis revealed that the fur-uncovered area was significantly decreased upon spermidine treatment (Mann Whitney test, *p* = 0.041; Fig. 1e). To exclude effects of barbering in aged animals, mice were separated into single cages at least 3 weeks prior the final experiments.

### Spermidine supplementation partly ameliorates age-related changes of brain glucose metabolism

Having observed that spermidine supplementation was effective against age-induced hair-loss, we next analysed whether 6 months of spermidine treatment would also have effects on another age-impaired functional entity - brain glucose metabolism. To investigate the cerebral effect of spermidine, we performed [^18^F]FDG PET in anaesthetised mice. We analysed three different parameters: (i) glucose uptake, (ii) the brain influx rate constant *K*_i_ and (iii) the metabolic rate of glucose uptake MR_Glu_. This was done in two different ways of image analysis: first, we used volume of interest (VOI) atlas-based approach, which allows for a comparison of functionally related brain areas within the treatment groups. Second, we used statistical parametric mapping (SPM)–based image analysis, which allows for statistical analysis of group differences at the atlas-independent whole-brain voxel-level.

**Fig. 2:**
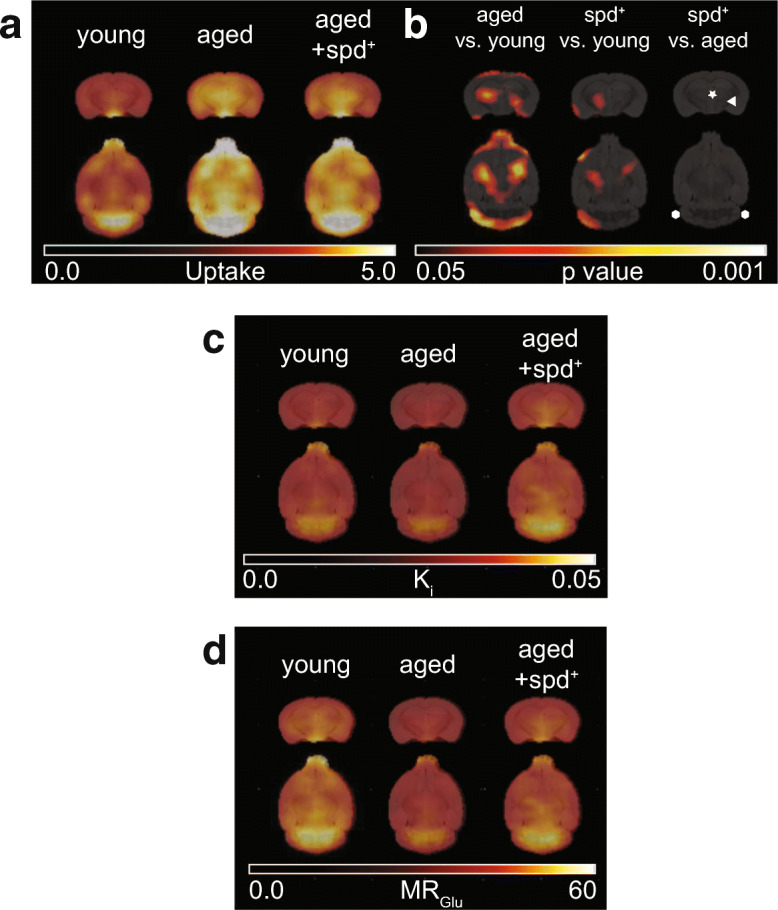
Coronal and horizontal average [^18^F]FDG PET parametric brain images of **a** [^18^F]FDG uptake ([%ID/cc]), **c** influx rate constant *K*_i_ [ml/g/min] and **d** glucose metabolic rate MR_Glu_ [μmol/min/100 g] calculated using Patlak graphical analysis in 6 month old (young) and in 23 month old untreated (aged) or spermidine-treated (aged +spd^+^) mice. **b** Results of voxel-based statistical parametric mapping of [^18^F]FDG uptake (unpaired 2-sample *t* test) identifying differences between young, aged and aged spd^+^ mice. The caudate putamen is indicated by a white arrowhead, thalamic regions by an asterisk. Cerebellum is flanked by two white hexagons. Threshold has been set to show only statistically significant voxels (*p* < 0.05; minimum cluster size of 50 voxels). Increases are indicated as hot scale. Additional data are presented in Supplementary Fig. S1

Analysing glucose uptake, which corresponds to the absolute amount of [^18^F]FDG in the brain in a certain time window, both ways of image analysis revealed a higher [^18^F]FDG uptake in aged animals compared to young animals in parts of the caudate putamen, thalamic regions and cerebellum (Fig. 2a, b). Such an increase is surprising and maybe a homeostatic counteraction to compensate for the usual functional decline of the brain during ageing. However, age-associated increase in glucose uptake was much less pronounced in the spd^+^ group (Fig. 2b, S1A), suggesting that spermidine-treatment might be able to attenuate the normally occurring age-induced increase in glucose uptake.

Next, we analysed a kinetic parameter, the brain influx rate constant *K*_i_, which is defined as the unidirectional uptake rate constant that incorporates both net inward transport and trapping of the radiotracer in tissue. Only slight group-related differences were found (Fig. S1B, D). Notably, however, the lowest *K*_i_ values were always obtained in the aged non-treated group independent of the way of analysis (Fig. 2c, S1B), and spermidine-treated and non-treated aged mice regionally differed significantly in SPM analysis (Fig. S1D).

The last investigated parameter was the metabolic rate of glucose uptake MR_Glu_, which takes into account the rate constant as well as individual blood glucose levels and biochemical differences between natural d-glucose and [^18^F]FDG. This analysis revealed that young animals showed higher MR_Glu_ in comparison to both aged non-treated and aged spd^+^ groups in various brain regions (Fig. 2d, S1C and S1E), reaching significance for the hypothalamic region (aged vs. young: *p* = 0.012, aged + spd^+^ vs. young: *p* = 0.033).

### Spermidine treatment ameliorates age-related pathologies in the heart, the kidney and the liver

**Fig. 3:**
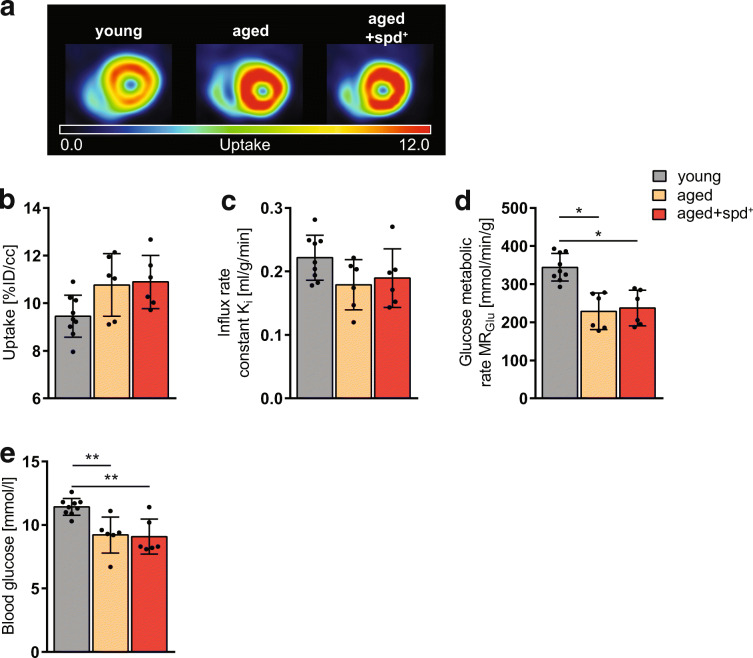
**a** Representative transaxial myocardial [^18^F]FDG PET uptake images in 6 month old young and in 23 month old untreated (aged) or spermidine-treated (aged+spd^+^) mice. Quantification of **b** myocardial [^18^F]FDG uptake [%ID/cc], **c** influx rate constant *K*_i_ [ml/g/min] and **d** glucose metabolic rate MR_Glu_ [μmol/min/100 g] in the left ventricular myocardium. **e** Blood glucose levels [mmol/l], measured prior to the PET scan. Significant differences calculated by one-way ANOVA and Tukey’s post hoc test comparing all groups with each other are indicated by asterisk (*p* < 0.05)

Ageing is frequently accompanied by cardiac disease (cardiac hypertrophy, myocardial infarction, etc.) leading to declined left ventricular function and ultimately heart failure. Therefore, we next investigated the impact of spermidine on heart function and heart tissue morphology in our aged mouse model. Analysing the left ventricular myocardium with [^18^F]FDG PET, we did not obtain any significant changes in [^18^F]FDG uptake and in *K*_i_ between groups (Fig. 3a to c). However, MR_Glu_ was significantly decreased in both aged groups compared to young animals (aged vs. young: *p* = 0.0002, aged + spd^+^ vs. young: *p* = 0.0004), whereas no difference between the two aged groups could be observed (Fig. 3d). While blood glucose values did not differ between aged and aged spd^+^ mice (Fig. 3e), young animals had significantly higher blood glucose levels (measurements before the [^18^F]FDG PET/CT scan) than either aged non-treated (*p* = 0.003) or aged spd^+^ mice (*p* = 0.005). These data suggest that an age-induced decrease in general glucose metabolism is not responsive to spermidine supplementation.

**Fig. 4:**
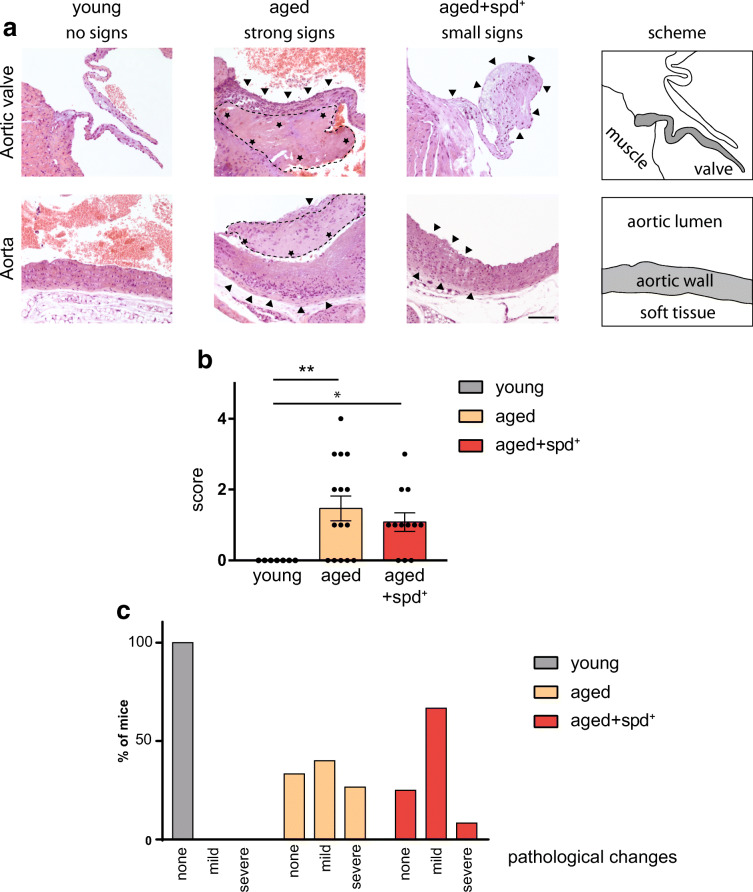
**a** Representative haematoxylin/eosin stainings of the histological heart samples. Upper panel illustrates pathophysiological changes within the aortic valve across all groups. The lower panels highlight the severity of different age-related changes within the aorta. Arrowheads point to tissue infiltration of macrophages characteristic for inflammation and tissue degeneration, whereas asterisks mark thrombus (dashed line) built-up within the aorta. Scale bar 100 μm. The scheme on the right illustrates anatomical structures analysed. Grey part undergoes pathological changes in aged mice and is shown in all images. **b** The pathological score (heart) as mean ± SEM. Kruskal-Wallis test with Dunn’s multiple comparison, adjusted p** = 0.010; p* = 0.039. *N* = 7 for young control, *N* = 15 for aged non-treated animals and *N* = 12 for spd-treated mice. **c** Histogram showing percentage of mice carrying none, mild or severe pathological heart changes

Having performed non-invasive molecular heart imaging, we then addressed the possible role of spermidine in preventing age-induced histological abnormalities within aged hearts (Fig. 4a). In this study, we mainly focused on inflammatory cardiac lesions, since lesions like calcification or myocardial infarction were not observed in any animal. As shown in Fig. 4b, the young control group showed no signs of age-related histo-pathology. Quantification of a histo-pathological/inflammatory score retrieved a significant increase in the aged non-treated and aged spd^+^ groups when compared to the young mice (Kruskal-Wallis test *p* = 0.010 and *p* = 0.039 respectively, Fig. 4b). To better visualize the effects of spermidine supplementation in ageing hearts, Fig. 4c shows the histogram of individual scores for different experimental groups (Fig. 4c). This histogram indicates that within the spermidine treated group, 8.3% of the mice suffered from severe inflammatory changes within the heart compared to 26.7% in the aged control group. Furthermore, in the treated group, 66.7% of the animals showed mild signs of inflammation, whereas in the aged control group only 40% showed mild changes, supporting a general beneficial effect of spermidine on the ageing heart.

Of note, none of the young mice showed neoplastic changes, whereas in 44% of the aged non-treated mice neoplastic changes occurred. In the aged spd^+^ mice, a similar number (37.5%) showed neoplastic changes, suggesting that spermidine treatment did not influence the occurrence of neoplasia.

In addition, we performed a histo-pathological analysis of the liver and the kidney using literature-based scores to quantify the severity of pathological changes. Within the liver, we recognised granuloma within the parenchyma in all groups as well as infiltrations within the bile duct to a similar extend ([65] and Fig. S2). Aged control and spermidine treated animals showed 33.3% and 37.5% severe pathological changes in the liver, respectively. Notably, however, necrotic changes within the liver were obtained only within the control aged group (11.1%), but not in the spermidine-treated one. Moreover, the percentage of animals with mild pathological changes was higher in the spermidine-treated group (62.5% vs. 55.6%).

In the kidney, we observed chronic nephropathy with few foci of basophilic tubules, no or mild interstitial mononuclear accumulation and some proteinaceous casts within both aged groups (Fig. S3). Furthermore, aged animals showed signs of a glomerulonephritis with some glomeruli affected by hypertrophy and hyperplasia of mesangial cells and dilatation of glomerular urinary space. Detailed histological analysis revealed that only 12.5% of spermidine treated animals show severe pathological changes compared to 33.3% in the aged group. At the same time, spermidine treatment increased the percentage of mildly affected animals up to 87.5%, while 66.7% of the aged control animals were mildly affected.

### Spermidine treatment protects against telomeres shortening in cardiac tissue

To test whether cardioprotective effects of spermidine were also associated with a modulation of telomere length in cardiac tissue, we applied quantitative fluorescence in situ hybridization (qFISH) to assess the relative telomere length in heart tissue sections [7].

**Fig. 5:**
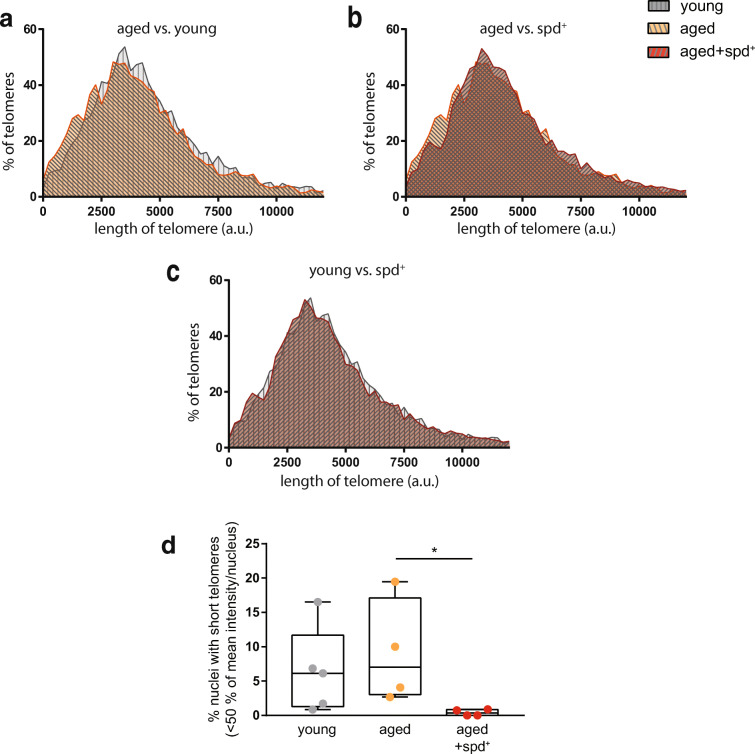
Comparison of the frequency distribution histograms of the telomere length between **a** non-treated aged mice and young mice, **b** non-treated aged and aged+spd^+^ mice and **c** aged+spd^+^ mice and young mice. At least 4.400 individual telomere signals per group were evaluated. **d** Quantification of the nuclei with short telomeres. Min-to-max box whisker plot is shown. Telomeres were defined as short, if their signals were below 50% of the mean telomere value of the same mouse. Each mouse is represented as a single dot in the plot. Kruskal-Wallis test with Dunn’s multiple comparison, adjusted *p* = 0.033

Frequency distribution analysis of the telomere length of at least 4.400 individual telomere signals per group revealed an expected shift to shorter telomeres in non-treated old animals in comparison to young mice (Fig. 5a). Intriguingly, a similar distribution was obtained when non-treated aged (more short telomeres) and aged spd^+^ (less short telomeres) groups were compared (Fig. 5b). Importantly, however, this ageing-related shift was absent when young and old spermidine-treated mice were compared (Fig. 5c). These differences are further underpinned by quantifying Spearman correlation coefficients for the different groups, which highlights the similarity between young and spermidine-treated groups (*R* = 1 vs. *R* = 0.97 for young vs. aged control) with regard to their telomere length distribution (Table 1).

**Table 1:** Spearman correlation coefficient between different groups highlighting the similarity between young and spermidine-treated groups with regard to telomere length distribution

Spearman coefficient [*R*]		*p* value
Young vs. control	0.97	1.48·10^−7^
Young vs. spd^+^	1.00	4.59·10^−11^
spd^+^ vs. control	0.97	1.97·10^−7^

**Fig. 6:**
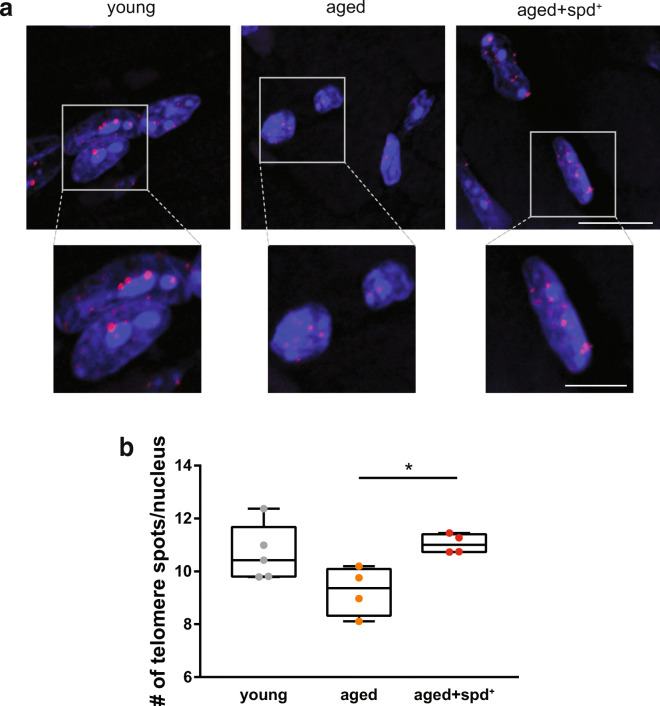
**a** Representative qFISH staining of paraffin heart sections for all three conditions. Telomeres were stained with Cy3 (red), and nuclei with DAPI (blue). Scale bar 10 μm (inset 5 μm). **b** Quantitative analysis of the number of detectable telomere signals per nucleus (*n* ≤ 120 nuclei/mouse). Kruskal-Wallis test with Dunn’s multiple comparison, adjusted *p* = 0.033

It is well documented that the percentage of short telomeres per cell is particularly associated with ageing and age-related pathologies including those of the heart [3, 61]. Thus, we determined the incidence of short telomeres for individual animals in the different experimental groups. Telomeres were defined as short if their signals were below 50% of the mean telomere length/nucleus value (defined individually for each animal). Strikingly, this analysis revealed that spermidine treatment provoked a significantly decreased percentage of nuclei with short telomeres in comparison to untreated aged animals (0.4% ± 0.47% in aged spd^+^ vs. 9.1% ± 7.63% in aged control; Kruskal Wallis test, *p* = 0.033, Fig. 5d). In addition, image analysis clearly demonstrated that nuclei of cardiac cells from young and aged spd^+^ animals contained more qFISH spots of high fluorescence intensity than nuclei from control old animals (Fig. 6a). These results were further corroborated by analysing the number of detectable telomere signals per nucleus. Here, numbers were significantly lower in aged controls when compared to aged spd^+^ mice, indicating that more telomeres had shortened below the detection limit in the aged mice under our specific experimental settings (Kruskal Wallis test, *p* = 0.033, Fig. 6b).

## Discussion

Spermidine supplementation previously has been shown to promote lifespan extension, although underlying mechanisms are not completely understood [34].

As ageing has multiple mechanistic dimensions, we investigated an extended spectrum of age-associated phenotypes in the present study. To this end, we compared young, normally aged and late-in-life spermidine supplemented aged wild-type mice in respect to several, translationally relevant age-related alterations.

By phenotypic analysis, we noticed that the ageing-related hair loss was decreased upon the long-term spermidine supplementation, suggesting a protective role of spermidine against hair loss (alopecia) in aged mice. Interestingly, these data are in line with in vitro findings that spermidine may promote human hair growth [47] and with clinical trial data indicating that spermidine supplementation may be beneficial against human hair loss [49]. These results further indicate that the spermidine supplementation per se was effective against age-induced deteriorations. Of note, in mice, denudation of special parts of the body can also be linked to a behavioural phenotype called barbering. It based on the interaction of a barber animal and a recipient in which the barber nibbles whiskers and fur of the recipient for various reasons [22]. To avoid a barbering, we separated mice into single cages at least 3 weeks prior to the final experiments. This time period should be enough for the fur regrowth [52], and thus, we think barbering does not play a role in the observed fur loss.

One possible reason for preserved fur growth in spd^+^ animals could be a spermidine-mediated induction of autophagy, which was documented across a wide spectrum of experimental models [18, 33, 36]. Indeed, previously, it was shown that quiescent hair follicles can be stimulated to initiate hair growth in mice by feeding small molecules activating autophagy, including α-ketoglutarate (α-KG) and α-ketobutyrate (α-KB) [8]. This is in line with the previous observation that supplementation of α-KB in old mice can prevent alopecia, which refers partial hair loss from head or body. A mechanistic link between autophagy and hair regeneration was further supported by the fact that stimulation of hair growth by α-KG and α-KB was blocked by specific autophagy inhibitors [8].

Alopecia is very common in human ageing [60]. Even tough not experimentally tested yet, one could thus speculate that dietary spermidine might increase hair growth in humans as well. Indeed, first evidence in this direction was obtained for human ex vivo hair follicle cultures, in which the spermidine-induced growth was shown to be autophagy-dependent [42]. Consistently, caloric restriction was shown to promote hair follicle growth as well, although the molecular mechanisms were not investigated in detail [17]. Since spermidine not only is an inducer of autophagy [44] but also has features of a caloric restriction mimetic [35], it could be interesting to explicitly test the protective potential of spermidine supplementation towards age-induced alopecia in future preclinical and clinical studies.

Of note, the spermidine-treated animals showed no increase in neoplastic changes compared to the aged non-treated animals, confirming that the prolonged supplementation with spermidine is safe in terms of neoplasia induction. This might be linked to spermidine-triggered depletion of regulatory T-cells [45]. This finding is also in line with our recent observations from a tolerability study which demonstrated that spermidine is well tolerated and does not increase morbidities or change behaviour in BALBc/Rj mice [53]. Furthermore, this study along others [12] showed that even though spermidine is proposed to harbour caloric restriction mimicking properties, food consumption usually does not change with the treatment, neither in young, nor in aged mice.

[^18^F]FDG PET was used to obtain novel information about spermidine effects on brain glucose metabolism. [^18^F]FDG PET is one of the commonly used non-invasive approaches to study functional decline in the ageing brain, with [^18^F]FDG as the most popular radiotracer for analysis of cerebral glucose uptake [6]. Particularly, neurodegenerative diseases are often accompanied with gluco-metabolic changes. While atlas-based uptake analysis did not result in significant changes, the statistical parametric mapping (SPM) approach, which allows for statistical comparison between the experimental groups, revealed that [^18^F]FDG uptake is significantly increased in caudate putamen, thalamus and cerebellum of aged animals when compared to young mice. Of note, this age-induced increase was less prominent in the aged + spd^+^ group. The reduced glucose uptake observed in young animals could be explained by the fact that [^18^F]FDG, which was administered in tracer dosage, is competing with endogenous blood glucose for brain uptake, with young mice being known to have higher blood glucose levels than old animals [28], as also confirmed in the present study.

In contrast to sole uptake analysis, kinetic modelling describes the dynamic tissue uptake relative to the plasma activity by measuring rate constants and therefore allows calculating values describing glucose turnover, an entity coupled with synaptic activity. Voxel-based average MR_Glu_ and *K*_i_ maps hint towards lower values in aged control mice but preserved or increased values in spermidine-treated animals compared to young mice. Accordingly, SPM analysis of *K*_i_ maps revealed significant regional differences between spermidine-treated vs. non-treated aged mice in cortical, thalamic and cerebellar brain tissue. SPM analysis of MR_Glu_ maps also displayed an age-dependent decrease in various brain regions, reaching significance in the hypothalamus. These finding underline the importance of performing kinetic modelling as sole uptake analysis without taking blood glucose into account can be misleading. As already mentioned, the metabolic rate of glucose consumption MR_Glu_ can be a measure reflecting synaptic activity. During physiological ageing, not all brain areas are affected to a similar extent. Indeed, there are hot spots of ageing within the brain with decline typically starting in thalamic areas [57]. Another brain region that is strongly affected during ageing is the hypothalamus [25]. Our PET analysis revealed ageing-related decrease in the metabolic rate within these brain areas in non-treated age mice, while spermidine supplementation seems to be protective against such changes. Among several pathways affected by spermidine, the observed changes might be explained by an improved mitochondrial function in these brain areas and by modulation of the synaptic vesicle pool [36].

Ageing is undoubtedly linked to inflammatory processes, which also cause many comorbidities. This scenario referred to as ‘inflamm-ageing’ plays a crucial role in the development of cardiovascular diseases [29]. In the present study, we found severe tissue inflammation within aortic valves and aortas in aged control animals, but not in the heart tissues of young mice. More importantly, these pathological changes were ameliorated upon spermidine supplementation, suggesting that spermidine possesses an anti-inflammatory effect [26]. Notably, previous analysis using a similar study design showed that spermidine supplementation reduced plasma TNF-α, and could thus contribute to a decrease of inflammation in heart tissue [12]. Furthermore, spermidine might improve cardiac health by facilitating mitochondrial biogenesis [64]. It is also noteworthy that our results suggest protective actions of spermidine in other organs as well, including the liver and the kidney. Consequently, this calls for future extended mouse studies in various ageing tissue types.

To investigate molecular details of spermidine action on cardiac tissue, we addressed the effects of ageing and spermidine interventions in respect to telomere length. Age-induced changes on molecular levels include accelerated telomere shortening, which was described across species borders, including mice and humans [61]. Telomere length represents a well-established biomarker to score the degree of age-induced deterioration, also for the cardiovascular system [14]. Consequently, preservation of telomere length by means of cardiac telomerase gene therapy was shown to confer cardioprotection in aged mice [4]. Importantly, it was previously demonstrated that supplementation of spermidine reduces cardiac hypertrophy and preserves diastolic function in aged mice [12], which is also supported by our own data. The length of telomeres in cardiomyocytes is basically not determined by cell division [9], but still undergoes pronounced shortening during ageing [58]. Our data clearly demonstrate that even when applied late-in-life (months 18 to 24), spermidine treatment was able to rescue age-related telomere shortening to levels comparable of young mice. Importantly, in our study, late-in-life spermidine supplementation could clearly decrease the amount of nuclei with short telomeres in cardiac tissues, known to be critical factors and indicators of cellular senescence and the development of cardiac pathologies [67]. The mechanisms by which spermidine supplementation might lead to less telomere attrition remains elusive at this point. Polyamines are known to play a crucial role in DNA stabilisation and might support G-quadrupole telomere in DNA structures [55], potentially interfering with telomere shortening. In addition, caloric restriction has been linked to attenuated telomere erosion in mice [62]. Recent research supports a beneficial effect of long telomeres, which go along with slowed metabolic ageing [41].

Another intriguing mechanism might involve spermidine-mediated protection of telomeres from oxidative stress. It has been widely documented that age-related increase in cardiac diseases is accompanied by increased level of oxidative stress mediated by reactive oxygen species (ROS), such as free radicals, oxygen ions and peroxides [10, 50]. Indeed, ROS are considered as a risk factor for a wide range of cardiac diseases in elderly [5], although underlying molecular mechanisms are not yet completely understood. In vitro studies show that telomeric DNA is more susceptible to cleavage by ROS than non-telomeric sequences [63]. Moreover, oxidative stress inhibits telomerase function in vivo, leading to the facilitation of direct ROS-damaging effect on telomeres [1]. On the other hand, natural polyamines, including spermidine, have long been known to act as free radical scavengers [19] and thus protect DNA against oxidative damages [24, 48]. In addition to the direct effects of polyamines on ROS, they stimulate the expression of proteins essential to an effective antioxidant response, including superoxide dismutases, glutathione and catalases [51]. Therefore, increased amounts of longer telomeres obtained in spd^+^ aged animals might also be explained by the spermidine-mediated reduction of ROS. Whether spermidine partially acts on healthy lifespan expansion by affecting telomerase activity or directly protecting telomeres from attrition by e.g. affecting mitochondrial dysfunction strongly warrants further investigation.

## Conclusions

Spermidine is an endogenous natural substance, whose concentration declines with age and whose systemic availability can be enhanced by both nutritional regimes and supplements. Our study links cardio-protective effects of spermidine at the histological level with decreased telomere attrition in heart tissue. In addition, spermidine might modulate age-related changes of brain glucose metabolism and ameliorate number of pathological sights in kidney and liver. Since late-in-life external administration of spermidine protects against many age-associated maladies and seems safe in humans, clinical trials may investigate the possibility of its use as an age-protective strategy.

## Supplementary information

Supplemental Fig. 1 Atlas-based regional brain analysis of [^18^F]FDG PET in 6 month old (young) as well as in 23 month old spermidine-treated (aged spd^+^) and non-treated (aged) mice for (**a**) [^18^F]FDG uptake ([%ID/cc]). Kinetic modelling (two-tissue compartment model) was used to calculate (**b**) brain influx rate constant *K*_i_ [ml/g/min] and (**c**) glucose metabolic rate MR_Glu_ [μmol/min/100 g]. Significant differences calculated by one-way ANOVA and Tukey’s post hoc test comparing all groups with each other are indicated by asterisk (*p* < 0.05). CPu, caudate putamen; Ctx, cortex; Hip, hippocampus; Thal, thalamus; Hypo, hypothalamus; Amyg, amygdala; Cb, cerebellum. **d** and **e** show the respective results of voxel-based statistical parametric mapping (unpaired 2-sample *t* test) identifying differences between young, aged and aged spd^+^ mice. Threshold has been set to show only statistically significant voxels (*p* < 0.05; minimum cluster size of 50 voxels). Decreases are indicated in cold scale. (PNG 482 kb)

High resolution (EPS 5502 kb)

Supplemental Fig. 2 **a** Representative haematoxylin/eosin stainings of the histological liver samples. Upper panel illustrates pathophysiological changes i.e. micro abcesses within the liver across all groups. The lower panels highlight the infiltration of the bile duct. Scale bar 50 μm. **b** The pathological score (liver) as mean ± SEM. Kruskal-Wallis test with Dunn’s multiple comparison. **c** Histogram showing percentage of mice showing/suffering none, mild, severe or necrotic pathological liver changes. (PNG 3179 kb)

High resolution (EPS 128020 kb)

Supplemental Fig. 3 **a** Representative periodic acid - Schiff reaction (PAS) stainings of histological kidney samples. Upper panel illustrates pathophysiological changes within the glomerulus across all groups. Arrowheads point to thickened basement membrane, whereas asterisks mark a dilatation of the glomerular urinary space. The outlining dashed line surrounds the glomerulus. Scale bar 20 μm. The lower panel shows nephropathic changes visualised by haematoxylin/eosin staining within the kidney cortex. The arrowheads indicate foci of basophilic tubules and an irregular surface of the kidney. Scale bar 200 μm. **b** The pathological score (kidney) as mean ± SEM. Kruskal-Wallis test with Dunn’s multiple comparison, adjusted p*** = 0.002, * = 0.012. **c** Histogram showing percentage of mice showing/suffering none, mild or severe pathological kidney changes. (PNG 3000 kb)

High resolution (EPS 124060 kb)

## Data Availability

The data that support the findings of this study are available from the corresponding author upon reasonable request.
