# Supplementary figures and images for: Novel aspects of age-protection by spermidine supplementation are associated with preserved telomere length
Source: GeroScience. 2021 Jan 31;43(2):673–90. doi: 10.1007/s11357-020-00310-0 (PMC8110654; doi:10.1007/s11357-020-00310-0)

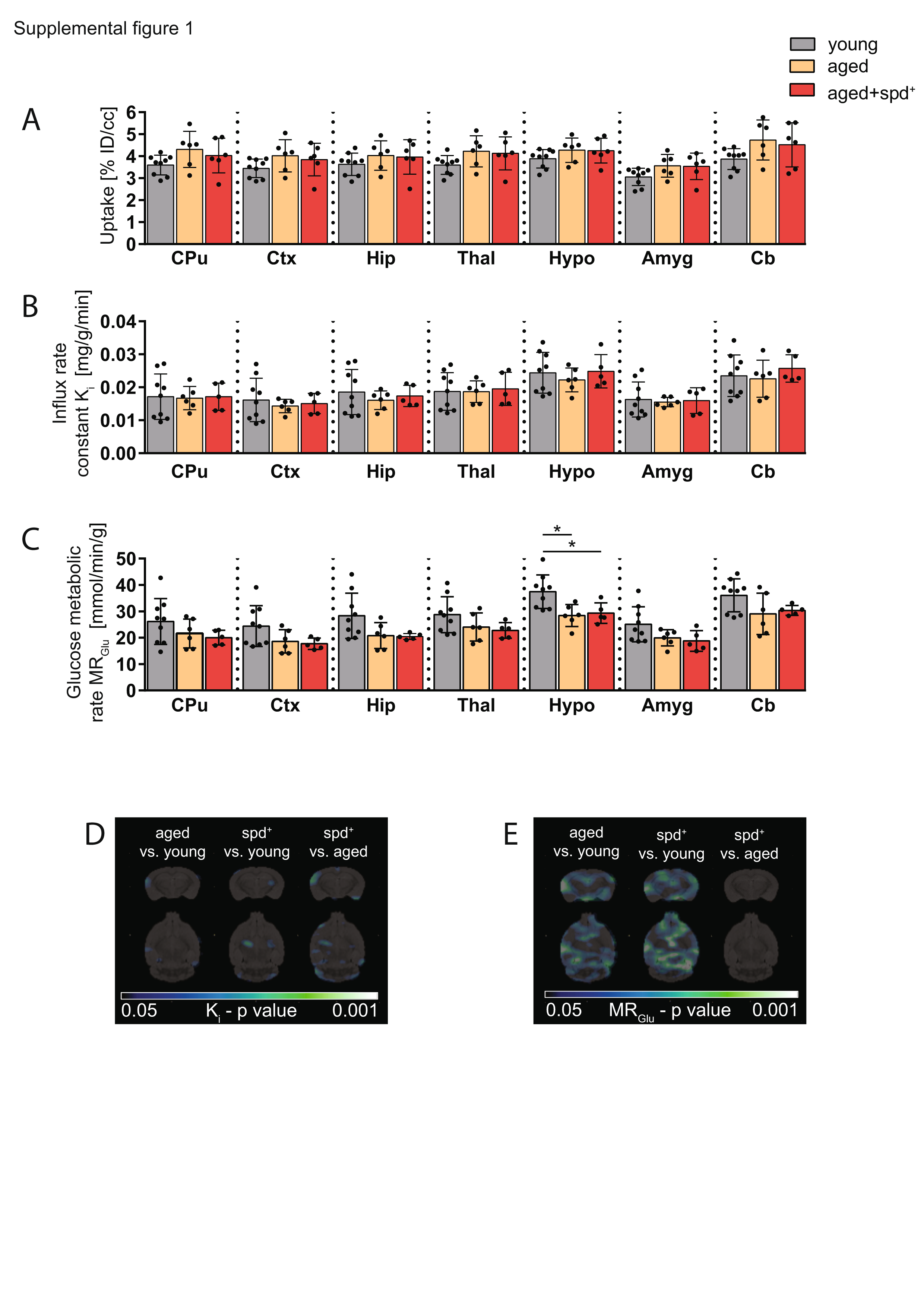

Supplement: Supplementary file 1 — Atlas-based regional brain analysis of [18F]FDG PET in 6 month old (young) as well as in 23 month old spermidine-treated (aged spd+) and non-treated (aged) mice for (a) [18F]FDG uptake ([%ID/cc]). Kinetic modelling (two-tissue compartment model) was used to calculate (b) brain influx rate constant Ki [ml/g/min] and (c) glucose metabolic rate MRGlu [μmol/min/100 g]. Significant differences calculated by one-way ANOVA and Tukey’s post hoc test comparing all groups with each other are indicated by asterisk (p < 0.05). CPu, caudate putamen; Ctx, cortex; Hip, hippocampus; Thal, thalamus; Hypo, hypothalamus; Amyg, amygdala; Cb, cerebellum. d and e show the respective results of voxel-based statistical parametric mapping (unpaired 2-sample t test) identifying differences between young, aged and aged spd+ mice. Threshold has been set to show only statistically significant voxels (p < 0.05; minimum cluster size of 50 voxels). Decreases are indicated in cold scale. (PNG 482 kb) [file 11357_2020_310_Fig7_ESM.png]

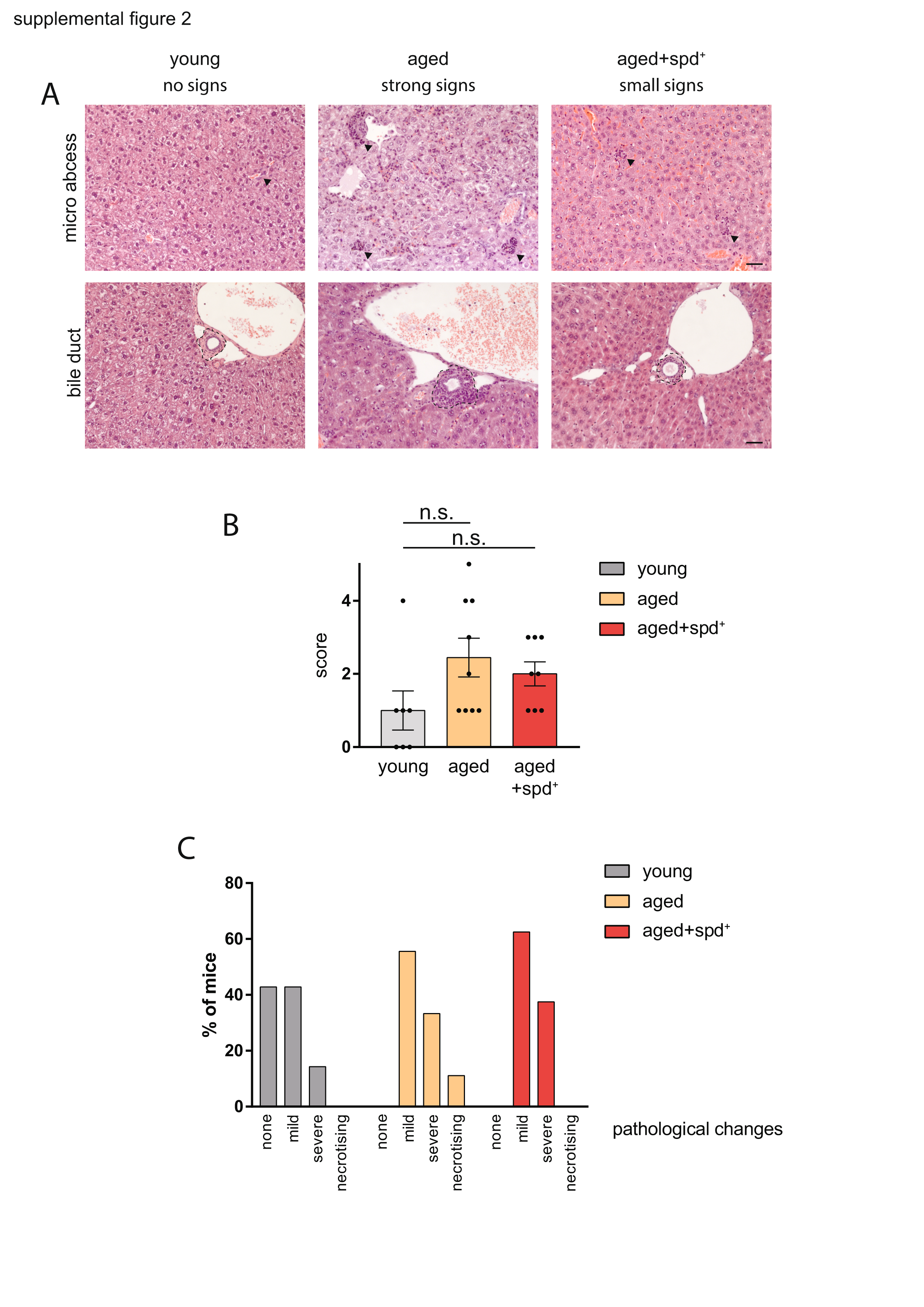

Supplement: Supplementary file 3 — a Representative haematoxylin/eosin stainings of the histological liver samples. Upper panel illustrates pathophysiological changes i.e. micro abcesses within the liver across all groups. The lower panels highlight the infiltration of the bile duct. Scale bar 50 μm. b The pathological score (liver) as mean ± SEM. Kruskal-Wallis test with Dunn’s multiple comparison. c Histogram showing percentage of mice showing/suffering none, mild, severe or necrotic pathological liver changes. (PNG 3179 kb) [file 11357_2020_310_Fig8_ESM.png]

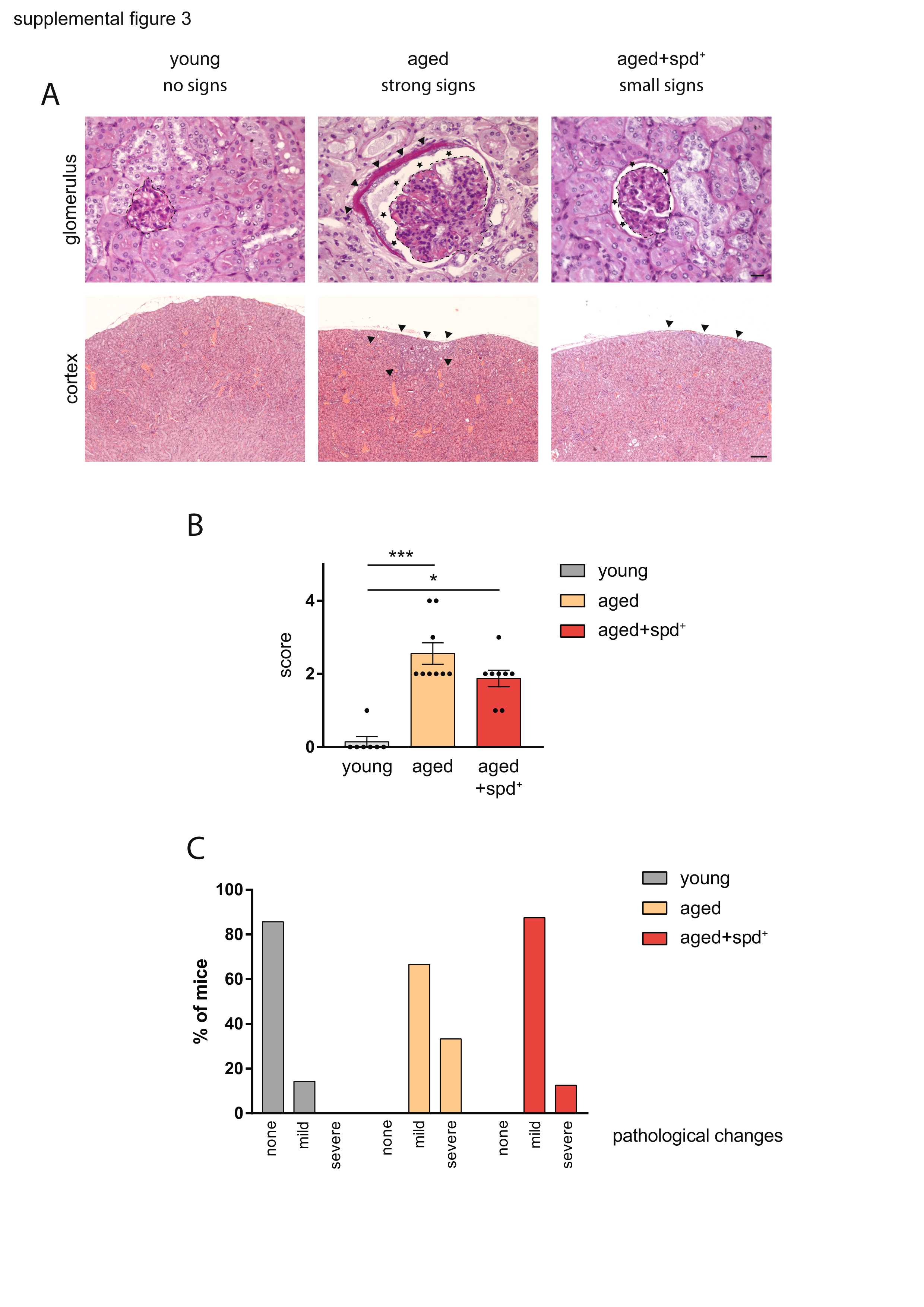

Supplement: Supplementary file 5 — a Representative periodic acid - Schiff reaction (PAS) stainings of histological kidney samples. Upper panel illustrates pathophysiological changes within the glomerulus across all groups. Arrowheads point to thickened basement membrane, whereas asterisks mark a dilatation of the glomerular urinary space. The outlining dashed line surrounds the glomerulus. Scale bar 20 μm. The lower panel shows nephropathic changes visualised by haematoxylin/eosin staining within the kidney cortex. The arrowheads indicate foci of basophilic tubules and an irregular surface of the kidney. Scale bar 200 μm. b The pathological score (kidney) as mean ± SEM. Kruskal-Wallis test with Dunn’s multiple comparison, adjusted p*** = 0.002, * = 0.012. c Histogram showing percentage of mice showing/suffering none, mild or severe pathological kidney changes. (PNG 3000 kb) [file 11357_2020_310_Fig9_ESM.png]
